# Impact of hypertension with or without diabetes on left ventricular remodeling in rural Chinese population: a cross-sectional study

**DOI:** 10.1186/s12872-017-0642-y

**Published:** 2017-07-27

**Authors:** Tan Li, Shuang Chen, Xiaofan Guo, Jun Yang, Yingxian Sun

**Affiliations:** 1 Department of Cardiovascular Ultrasound, the First Affiliated Hospital of China Medical University, 155 Nanjing North Street, Heping District, Shenyang, 110001 People’s Republic of China; 2 Department of Cardiology, the First Affiliated Hospital of China Medical University, 155 Nanjing North Street, Heping District, Shenyang, 110001 People’s Republic of China

**Keywords:** Hypertension, Diabetes, Left ventricular remodeling, Left ventricular geometry

## Abstract

**Background:**

The aim of this study was to assess the impact of hypertension with or without diabetes on left ventricular (LV) remodeling in rural Chinese population.

**Methods:**

A total of 10,270 participants were classified into control group, hypertension without diabetes (HT) group, and hypertension with diabetes (HT + DM) group. We compared clinical characteristics and echocardiographic parameters, and used multivariable logistic regression analysis to assess the associations of interest.

**Results:**

HT + DM group had higher interventricular septal thickness (IVSd), posterior wall thickness (PWTd), left ventricular mass (LVM), LVM index (LVMI), relative wall thickness (RWT), left atrial diameter (LAD), A wave and lower E wave than HT group (all *P* < 0.05). The prevalence rates of left ventricular hypertrophy (LVH) and abnormal geometry were statistically different among three groups (*P* < 0.001) and eccentric hypertrophy was the highest proportion of geometry abnormality. Logistic regression analysis suggested that subjects in HT and HT + DM groups had odds ratio (OR) values of 2.81, 4.41, 2.24 and 3.94, 7.20, 2.38 for LVH, concentric hypertrophy and eccentric hypertrophy in the total population, respectively, compared to control group. When compared with HT group, those in HT + DM group had approximately 1.40-, 1.61- and 1.38-, 1.71-fold increased risk for LVH and concentric hypertrophy in the total and female population separately, but no association of HT + DM with LVH and abnormal geometrical patterns was found in men.

**Conclusions:**

This study demonstrated that, to varying degrees, hypertension was associated with LV remodeling in rural Chinese population, and this risk association was obviously increased for LVH and concentric hypertrophy when accompanied by diabetes, especially for women.

## Background

Among hypertensive patients, left ventricular hypertrophy (LVH) and abnormal geometry are both important markers of cardiac remodeling strongly associated with cardiovascular risk and prognosis [1]. There are three abnormal LV geometrical patterns including concentric remodeling, concentric hypertrophy and eccentric hypertrophy [2]. The various prevalence rates of LVH and abnormal geometrical patterns have been described in different hypertensive populations, but which type is predominated is controversial [3–5]. Alterations in cardiac metabolism for example are of particular interest in diabetes, where an increased reliance on fatty acid oxidation and uncoupling between glucose oxidation and glycolysis are suggested to promote LVH [6]. Hypertension coexisting with diabetes therefore can further increase the risk of target organ injury and clinical cardiovascular accident [7]. However, their combined and sex-specific effects on LV remodeling remain unclear.

Investigators have reported that patients with hypertension have high prevalence of LV remodeling or geometrical changes [8, 9], but data concerning the cardiac features of hypertensive subjects with or without diabetes remain incompletely described in large population-based samples, especially in the rural population of China, where hypertension is often popular. Therefore, we conducted this cross-sectional study to investigate the impact of hypertension with or without diabetes and its possible gender-dependent effect on LV remodeling, hypertrophy and geometry in northeast rural Chinese population.

## Methods

### Study population

Detail methods were described in previous studies [10–13]. From January 2012 to August 2013, a representative sample of individuals aged ≥35 years was chosen to present the prevalence, incidence and natural history of cardiovascular risk factors in rural areas of Liaoning Province, located in Northeast China. We conducted this cross-sectional study using a multi-stage, stratified randomly cluster-sampling scheme. In the first stage, three counties (Dawa, Zhangwu, and Liaoyang County) were selected from the eastern, southern, and northern region of Liaoning province. In the second stage, one town was randomly chosen from each three counties. In the third stage, 8–10 rural villages from each town were randomly selected (a total of 26 rural villages). All the eligible permanent residents aged ≥35 years from each village were invited to attend the study (a total of 14,016 participants). Of those, 11,956 participants (i.e. response rate of 85.3%) agreed and completed the study. The study was approved by the Ethics Committee of China Medical University (Shenyang, China). All procedures were performed in accordance with the ethical standards. Written consent was obtained in all participants after they had been informed of the objectives, benefits, medical items and confidentiality agreement of personal information. If the participants were illiterate, we obtained the written informed consents from their proxies. In this report, subjects were excluded if they met any of the following criteria: pregnancy, malignant tumor, mental disorder, congenital heart disease, myocardial infarction, pericardia disease, cardiomyopathy or other organic heart disease, all kinds of serious arrhythmia, heart valve disease (the valve with moderate to severe reflux or stenosis) and diabetes without hypertension. Our final sample consisted of 10,270 participants.

### Data collection and measurements

The data collection and measurements used in this study were described in previous studies [10, 12–14]. Data were collected via a single visit by experienced cardiologists and trained nurses, where a standard questionnaire was used by a face-to-face interview. Before the survey was conducted, we called for all eligible investigators to undergo the organized training on the purpose of this study, how to administer the questionnaire, the standard method of measurement, the importance of standardization, and the study procedures. A strict test was performed at the end of this training, and those who scored perfectly on the test could become investigators. During data collection, our inspectors provided further instructions and support. A central steering committee with a subcommittee for quality control was organized.

Weight and height were measured to the nearest 0.1 kg and 0.1 cm respectively with the participants in light weight clothing and without shoes. Waist circumference (WC) was measured at the umbilicus using a non-elastic tape (to the nearest 0.1 cm) in standing position at the end of normal expiration. Body mass index (BMI) was calculated as weight (kg)/height^2^ (m^2^). Body surface area (BSA) was calculated as [0.0061 × height (cm) +0.0128 × weight (kg)-0.1529].

According to American Heart Association protocol, blood pressure was measured 3 times at 2-min intervals after at least 5 min of rest. Blood pressure measurements were acquired with a standardized automatic electronic sphygmomanometer (HEM-907; Omron) validated by the British Hypertension Society protocol. The participants were asked to avoid caffeinated beverages and exercise for at least 30 min before the measurement. During the measurement, participants were seated with the arm supported at the level of the heart. The mean of three BP measures was calculated for analysis. Hypertension was defined as systolic blood pressure (SBP) ≥140 mmHg and (or) diastolic blood pressure (DBP) ≥90 mmHg and (or) being under antihypertensive treatment according to JNC-7 report [15].

Fasting blood samples were collected in the morning after at least 12 h of fasting for all participants. Blood samples were obtained from an antecubital vein into Vacutainer tubes containing EDTA. Total cholesterol (TC), triglyceride (TG), low-density lipoprotein cholesterol (LDL-C), high-density lipoprotein cholesterol (HDL-C), fasting plasma glucose (FPG), plasma creatine (PC), hemoglobin and uric acid (UA) were analyzed enzymatically on an autoanalyzer. All laboratory equipment was calibrated, and blind, duplicate samples were used. Diabetes was defined as FPG ≥ 7 mmol/L (126 mg/dL) and/or being on treatment for diabetes according to the WHO criteria [16].

### Echocardiography

Echocardiographic examination was described in our previous study [10]. The echocardiograms were obtained using a commercially available Doppler machine (Vivid, GE Healthcare, United States), with a 3.0 MHz transducer using M-mode, 2-dimensional, spectral and color Doppler transthoracic echocardiography. Echocardiogram analyses and readings were performed by three physicians majored in echocardiography.

According to the guideline of the American Society of Echocardiography [17], the parasternal long-axis view was measured to record interventricular septal thickness (IVSd), LV end-diastolic internal dimension (LVIDd), posterior wall thickness (PWTd) at end-diastole, and LV end-systolic internal dimension (LVIDs), left atrial diameter (LAD), aortic annular diameter (AOD) during systole. The mitral inflow velocities were obtained by pulsed wave Doppler at the level of mitral valve tips in the apical four-chamber view, and early diastolic peak flow (E), atrial peak flow (A) and E/A ratio were recorded. The LV end-diastolic volume (LVEDV) and LV end-systolic volume (LVESV) were estimated by Teichholz eqs. [18]: LVEDV (ml) = LVIDd^3^ × 7.0/(2.4 + LVIDd), LVESV (ml) = LVIDs^3^ × 7.0/(2.4 + LVIDs). LV ejection fraction (EF) was calculated as [(LVEDV-LVESV)/LVEDV] × 100% and fractional shortening (FS) was determined using [(LVIDd-LVIDs)/LVIDd] × 100%.

Left ventricular mass (LVM) was estimated according to the formula: LVM = 0.8× [1.04{(IVSTd + PWTd + LVIDd)^3^–LVIDd^3^}] + 0.6 g. LVM was divided by height^2.7^ to obtain left ventricular mass index (LVMI). LVH was diagnosed when LVMI was >48 g/ht^2.7^ in men and >44 g/ht^2.7^ in women [19, 20]. The relative wall thickness (RWT) = (IVSd + PWTd)/LVIDd and considered increased if > 0.42 [20]. LV geometry was assessed from LVMI and RWT values [9, 20], including normal geometry with normal LVMI and normal RWT, concentric remodeling with normal LVMI and increased RWT, concentric hypertrophy with increased LVMI and increased RWT, and eccentric hypertrophy with increased LVMI and normal RWT.

### Statistical analysis

Descriptive statistics were calculated for all the variables. Continuous variables were reported as mean ± standard deviations, and categorical variables were reported as numbers and percentages. Differences among categories were evaluated using ANOVA, non-parametric test or the χ2-test as appropriate. Multivariable logistic regression analyses were used to identify the associations of HT, HT + DM with LVH and abnormal geometrical patterns by calculating odds ratios (ORs) and 95% confidence intervals (CIs). All statistical analyses were performed using SPSS version 17.0 software, and *P*<0.05 indicated statistical significance.

## Results

### Clinical characteristics

Of the 10,270 participants, there were 4990 (48.6%) subjects without hypertension and diabetes (control group), 4462 (43.4%) subjects with hypertension without diabetes (HT group) and 818 (8.0%) subjects with hypertension and diabetes (HT + DM group). The clinical characteristics of participants in each group were described and compared in Table 1. Participants in HT + DM group tended to have higher weight, WC, BSA, BMI, SBP, TC, TG, LDL-C and FPG than control and HT groups.

**Table 1:** The clinical characteristics of participants in each group

Variables	Control	HT	HT + DM
	*N* = 4990	*N* = 4462	*N* = 818
Age	49.9 ± 9.4	56.9 ± 10.3^*^	58.3 ± 9.0^#†^
Males, n (%)	2146 (43.0%)	2200 (49.3%)	353 (43.2%)^**^
Han ethnicity, n (%)	4743 (95.1%)	4236 (94.9%)	778 (95.1%)
Height, cm	161.0 ± 8.0	160.3 ± 8.3^*^	159.7 ± 8.4^#^
Weight, kg	62.2 ± 10.7	65.4 ± 11.5^*^	67.8 ± 11.7^#†^
WC, cm	79.6 ± 9.2	84.3 ± 9.6^*^	87.7 ± 9.3^#†^
BSA, m2	1.62 ± 0.17	1.66 ± 0.18^*^	1.69 ± 0.19^#†^
BMI, kg/ m^2^	23.94 ± 3.49	25.39 ± 3.62^*^	26.49 ± 3.65^#†^
BMI ≥ 30 kg/ m^2^, n (%)	237 (4.7%)	423 (9.5%)	131 (16.0%)^**^
Current smoke, n (%)	1761 (35.3%)	1603 (35.9%)	231 (28.2%)^**^
Current drinking, n (%)	1015 (20.3%)	1114 (25.0%)	177 (21.6%)^**^
Heart rate, bmp	76.36 ± 11.66	77.95 ± 13.42^*^	85.65 ± 14.99^#†^
SBP, mmHg	123.70 ± 9.83	158.06 ± 18.97^*^	162.11 ± 20.57^#†^
DBP, mmHg	74.96 ± 7.37	88.75 ± 10.85^*^	88.89 ± 11.60^#^
TC, mmol/L	5.03 ± 1.00	5.39 ± 1.09^*^	5.69 ± 1.24^#†^
TG, mmol/L	1.39 ± 1.20	1.69 ± 1.29^*^	2.59 ± 2.62^#†^
LDL-C, mmol/L	2.76 ± 0.74	3.06 ± 0.84^*^	3.23 ± 0.94^#†^
HDL-C, mmol/L	1.40 ± 0.36	1.44 ± 0.41^*^	1.32 ± 0.35^#†^
FPG, mmol/L	5.42 ± 0.52	5.60 ± 0.56^*^	9.30 ± 3.14^#†^
PC, mg/dl	70.82 ± 12.24	72.89 ± 28.58^*^	72.74 ± 19.16^#^
Hemoglobin, g/dl	136.70 ± 19.56	140.46 ± 18.46^*^	141.52 ± 16.23^#^
UA, umol/L	280.38 ± 80.08	300.70 ± 86.40^*^	302.26 ± 91.51^#^
Antihypertensive medication, n (%)	0.00	1195 (26.8%)	330 (40.3%)^**^
Antidiabetic medication, n (%)	0.00	0.00	312 (38.1%)^**^

*HT* hypertension without diabetes, *HT + DM* hypertension with diabetes

^*^ *P* < 0.05 between HT and Control, ^#^ *P* < 0.05 between HT + DM and Control, ^†^ *P* < 0.05 between HT + DM and HT, ^**^ *P* < 0.001 among three groups

### Echocardiographic parameters comparison

As shown in Table 2, compared to control group, IVSd, LVIDd, LVIDs, PWTd, LVM, LVMI, RWT, AOD, LAD/BSA, A wave, LVEDV, LVESV and SV were significantly higher, while E wave was lower in HT and HT + DM groups (*P* < 0.05). HT + DM group had higher IVSd, PWTd, LVM, LVMI, RWT, LAD, A wave and lower E wave than HT group (*P* < 0.05). The prevalence of LVH was statistically different in the three groups (*P* < 0.001) and eccentric hypertrophy was the highest proportion of geometry abnormality. The prevalence rates of eccentric hypertrophy, concentric remodeling and concentric hypertrophy were statistically different among groups. Furthermore, we compared the echocardiographic parameters of participants based on age groups. The results showed that, as age increased, IVSd, LVIDd, LVIDs, PWTd and A wave became higher, while E wave and E/A became lower from 35 to 45 year group to >65 year group (*P* < 0.05).

**Table 2:** Echocardiographic parameters comparison

Variables	Control	HT	HT + DM
	*N* = 4990	*N* = 4462	*N* = 818
IVSd, cm	0.84 ± 0.15	0.92 ± 0.30^*^	0.94 ± 0.25^#†^
LVIDd, cm	4.64 ± 0.39	4.76 ± 0.42^*^	4.76 ± 0.45^#^
LVIDs, cm	3.05 ± 0.39	3.15 ± 0.43^*^	3.16 ± 0.43^#^
PWTd, cm	0.83 ± 0.29	0.89 ± 0.21^*^	0.92 ± 0.28^#†^
LVM, g	123.00 ± 73.62	151.87 ± 94.89^*^	159.60 ± 139.43^#†^
LVMI, g/m^2.7^	35.98 ± 20.67	42.49 ± 25.77^*^	45.03 ± 36.37^#†^
RWT	0.36 ± 0.08	0.38 ± 0.09^*^	0.39 ± 0.08^#†^
LVH, n (%)	369 (7.4%)	1209 (27.1%)	293 (35.8%)^**^
AOD, cm	2.20 ± 0.30	2.27 ± 0.30^*^	2.28 ± 0.32^#^
LAD, cm	3.27 ± 0.36	3.44 ± 0.39^*^	3.49 ± 0.40^#†^
LAD/BSA, cm/ m^2^	2.03 ± 0.23	2.08 ± 0.26^*^	2.08 ± 0.26^#^
E wave, cm/s	75.90 ± 19.30	70.78 ± 18.90^*^	66.47 ± 18.13^#†^
A wave, cm/s	70.24 ± 15.64	80.22 ± 17.45^*^	86.71 ± 18.37^#†^
E/A	1.16 ± 1.43	1.03 ± 6.55	0.82 ± 0.67^#^
LVEDV, ml	100.35 ± 19.59	106.55 ± 22.08^*^	107.01 ± 26.26^#^
LVESV, ml	37.54 ± 11.80	40.67 ± 13.59^*^	40.82 ± 13.60^#^
SV, ml	62.81 ± 16.07	65.88 ± 18.42^*^	66.19 ± 22.79^#^
LVEF, %	0.62 ± 0.10	0.62 ± 0.10^*^	0.62 ± 0.10^#^
FS, %	0.34 ± 0.07	0.34 ± 0.07^*^	0.34 ± 0.08
Normal geometry, n (%)	4346 (87.1%)	2869 (64.3%)	445 (54.4%)^**^
Concentric remodeling, n (%)	275 (5.5%)	384 (8.6%)	80 (9.8%)^**^
Concentric hypertrophy, n (%)	60 (1.2)	432 (9.7%)	129 (15.8%)^**^
Eccentric hypertrophy, n (%)	309 (6.2%)	777 (17.4%)	164 (20.0%)^**^

*HT* hypertension without diabetes, *HT + DM* hypertension with diabetes

^*^ *P* < 0.05 between HT and Control, ^#^ *P* < 0.05 between HT + DM and Control, ^†^ *P* < 0.05 between HT + DM and HT, ^**^ *P* < 0.001 among three groups

### Hypertension with or without diabetes and LV geometry abnormality

Adjustment for age, gender, race, height, weight, WC, heart rate, smoking, drinking, TC, TG, LDL-C, HDL-C, PC, hemoglobin, UA,antihypertensive and antidiabetic medication, the logistic regression models showed that subjects in HT and HT + DM groups had OR values of 2.81, 4.41, 2.24 and 3.94, 7.20, 2.38 for LVH, concentric hypertrophy and eccentric hypertrophy in the total population, respectively, compared with control group. When compared with subjects in HT group, the total and female population in HT + DM group had approximately 1.40-, 1.61- and 1.38-, 1.71-fold increase in the risk of LVH and concentric hypertrophy separately, however, there existed no association of HT + DM with LVH and abnormal geometrical patterns in male population. Table 3.

**Table 3:** Multiple logistic regression analysis in HT and HT + DM groups for men and women

Classification	LVH		Concentric remodeling		Concentric hypertrophy		Eccentric hypertrophy	
	OR (95% CI)	*P*	OR (95% CI)	*P*	OR (95% CI)	*P*	OR (95% CI)	*P*
Total								
Control	1.00		1.00		1.00		1.00	
HT	2.81 (2.43–3.26)	<0.001^*^	1.32 (1.09–1.59)	0.004^*^	4.41 (3.28–5.92)	<0.001^*^	2.24 (1.90–2.64)	<0.001^*^
HT + DM	3.94 (3.08–5.03)	<0.001^*^	1.23 (0.86–1.75)	0.248	7.20 (4.91–10.55)	<0.001^*^	2.38 (1.80–3.16)	<0.001^*^
HT	1.00		1.00		1.00		1.00	
HT + DM	1.40 (1.12–1.75)	0.003^*^	1.01 (0.72–1.42)	0.953	1.61 (1.22–2.12)	0.001^*^	1.07 (0.83–1.39)	0.604
Men								
Control	1.00		1.00		1.00		1.00	
HT	2.93 (2.31–3.72)	<0.001^*^	1.71 (1.27–2.29)	<0.001^*^	4.66 (2.95–7.35)	<0.001^*^	2.27 (1.72–2.98)	<0.001^*^
HT + DM	4.34 (2.98–6.33)	<0.001^*^	1.83 (1.09–3.05)	0.022	6.67 (3.68–12.10)	<0.001^*^	2.98 (1.90–4.65)	<0.001^*^
HT	1.00		1.00		1.00		1.00	
HT + DM	1.41 (0.81–2.17)	0.153	1.07 (0.66–1.74)	0.777	1.42 (0.92–2.19)	0.116	1.25 (0.84–1.87)	0.274
Women								
Control	1.00		1.00		1.00		1.00	
HT	2.74 (2.26–3.31)	<0.001^*^	1.12 (0.87–1.43)	0.383^*^	4.11 (2.78–6.08)	<0.001^*^	2.24 (1.82–2.75)	<0.001^*^
HT + DM	3.68 (2.65–5.10)	<0.001^*^	0.95 (0.58–1.55)	0.836	7.21 (4.35–11.95)	<0.001^*^	2.11 (1.46–3.03)	<0.001^*^
HT	1.00		1.00		1.00		1.00	
HT + DM	1.38 (1.02–1.87)	0.035^*^	0.96 (0.60–1.55)	0.865	1.71 (1.18–2.45)	0.004^*^	0.98 (0.70–1.38)	0.898

Adjustment for age, gender, race, height, weight, WC, heart rate, smoking, drinking, TC, TG, LDL-C, HDL-C, PC, hemoglobin, UA,antihypertensive and antidiabetic medication

*HT* hypertension without diabetes, *HT + DM* hypertension with diabetes, *LVH* left ventricular hypertrophy

^*^ *P* < 0.05

## Discussion

In this study, we investigated the impact of hypertension with or without diabetes on LV hypertrophy, geometry and function in northeast rural Chinese population. Our results suggested that different degree of LV remodeling was found in HT and HT + DM groups. Although there were no significant differences in LVIDd, LVIDs, LVEDV, LVESV and SV that reflected ventricular volume indexes between HT + DM and HT group, the values of IVSd, PWTd, LVM, LVMI, RWT and LAD were significantly higher in HT + DM group than HT group. It indicated that in the presence of diabetes, hypertension could produce more obvious LV hypertrophy and remodeling. Diabetes is a well-established risk factor for developing cardiovascular disease [21] and diabetic hypertensive patients may have more severe clinical and morphologic features of cardiovascular disease compared to those with hypertension only [22, 23]. Recent studies in mice models further found that diabetes by itself had no overt effect on cardiac structural change, but that combined with hypertension, diabetic heart appeared to potentiate LV hypertrophic remodeling [24–26]. This point could help us suppose that the diabetic condition sensitized the heart to other common pathological factors, like hypertension. Impaired LV diastolic function, followed by a decrease in early-diastolic ventricular filling and a greater atrial contribution to ventricular filling in the late-systolic period, could appear much earlier than systolic dysfunction [27]. Our study showed that diabetic hypertensive patients had worse diastolic function with lower E wave and higher A wave than non-diabetic hypertensive patients.

The LV adaptation to hemodynamic changes takes four different geometric patterns using LVMI together with RWT, which is determined to a great degree by whether pressure or volume overload is predominating. In our study, about 35.7% of hypertensive subjects without diabetes and 45.6% of hypertensive ones with diabetes had abnormal geometrical changes, in which eccentric hypertrophy was the highest proportion. Eccentric hypertrophy may be the early cardiac presentation of a pathological process without an intervention of concentric hypertrophy [1] and its high prevalence may be associated with the status of volume overload which plays a critical role in LV adaptation in hypertension with or without diabetes [28, 29]. A common belief was that concentric remodeling or hypertrophy was the predominant type of abnormal LV geometry among hypertensive population [30–32]. On the contrary, data from some epidemiologic studies have indicated that eccentric hypertrophy was even more common than concentric hypertrophy in hypertensive population [33], which is similar to our finding. The differences in geometric patterns might be due to population heterogeneity, genetic variability, diagnostic criteria of LVH and risk factors [9, 34].

Furthermore, after multivariable adjustment, results from logistic regression analysis indicated a positive and significant association of LVH and three abnormal geometrical patterns with non-diabetic hypertension. Hypertension coexisting with diabetes has been shown to be correlated with concentric hypertrophy rather than eccentric hypertrophy [7, 35, 36]. However, in our study, when compared with control group, we demonstrated that hypertension with diabetes was not only significantly associated with concentric hypertrophy, but also with LVH and eccentric hypertrophy in the total, male and female population, respectively. Moreover, we found that subjects in HT + DM group had about 1.5-fold higher risk for LVH and concentric hypertrophy than those from HT group in the total and female population, but no such association for men. Thus, we further provided evidence that hypertension and diabetes had additive effects of pressure load and sugar metabolic disorder on LV hypertrophy and abnormal geometrical changes. Of note, hypertension had strong effects on LV remodeling, and an additional contribution of diabetes was obtained only in women but not in men [37]. Such a sex-specific difference might be contributed to the presence of sensitive estrogen receptors in cardiomyocytes and insulin resistance in diabetes counterbalancing the beneficial cardiovascular influences of estrogen in women [38]. Therefore, there is an urgent need to strengthen the control of hypertension with diabetes, especially for female patients, in order to avoid subsequent serious cardiovascular events and burden on the rural population.

Our study has some limitations. First, based on a cross-sectional design, we were unable to draw causal inferences exactly from the results. Second, although we have a strict operation and diagnostic criteria, small and inevitable differences in performance technique among doctors might influence results. Third, detailed and complete records of glycated hemoglobin (HBA1c) level, types of hypertensive drugs, dose and compliance were not available. Fourth, diagnosis of diabetes relied on FPG level and/or being on treatment for diabetes without integrating HbA1c into the diagnostic criteria, which suggests that the prevalence of hypertension with diabetes may have been underestimated [39].

## Conclusions

In summary, there existed LV remodeling in hypertensive subjects with or without diabetes in rural Chinese population, and eccentric hypertrophy was the commonest abnormal geometrical pattern. Hypertension was an independent risk factor for LVH and geometrical changes, and this risk was increased by different degrees when accompanied with diabetes. Compared to non-diabetic hypertension, hypertension with diabetes was more important for an increase in the risk of LVH and concentric hypertrophy in women, but not in men. Moreover, further prospective studies are required to understand the predictive usefulness of LVH and geometrical changes in risk assessment in these patient groups.

## Abbreviations

A: Atrial peak flow
AOD: Aortic annular diameter
BMI: Body mass index
BSA: Body surface area
DBP: Diastolic blood pressure
E: Early transmitral diastolic peak flow
EF: Ejection fraction
FPG: Fasting plasma glucose
FS: Fractional shortening
HDL-C: High-density lipoprotein cholesterol
IVSd: Interventricular septal thickness
LAD: Left atrial diameter
LDL-C: Low-density lipoprotein cholesterol
LV: Left ventricular
LVEDV: LV end-diastolic volume
LVESV: LV end-systolic volume
LVH: Left ventricular hypertrophy
LVIDd: LV end-diastolic internal dimension
LVIDs: LV end-systolic internal dimension
LVM: Left ventricular mass
LVMI: Left ventricular mass index
PC: Plasma creatine
PWTd: Posterior wall thickness
RWT: Relative wall thickness
SBP: Systolic blood pressure
TC: Total cholesterol
TG: Triglyceride
WC: Waist circumference
